# Identifying and extracting bark key features of 42 tree species using convolutional neural networks and class activation mapping

**DOI:** 10.1038/s41598-022-08571-9

**Published:** 2022-03-19

**Authors:** Tae Kyung Kim, Jeonghyun Hong, Daun Ryu, Sukyung Kim, Si Yeon Byeon, Woojin Huh, Kunhyo Kim, Gyu Heon Baek, Hyun Seok Kim

**Affiliations:** 1 Department of Agriculture, Forestry and Bioresources, Seoul National University, Seoul, 08826 Republic of Korea; 2 Department of Forest Sciences, Seoul National University, Seoul, 08826 Republic of Korea; 3 Interdisciplinary Program in Agricultural and Forest Meteorology, Seoul National University, Seoul, 08826 Republic of Korea; 4 National Center for Agrometeorology, Seoul, 08826 Republic of Korea; 5 Research Institute of Agricultural and Life Sciences, Seoul National University, Seoul, 08826 Republic of Korea

**Keywords:** Information technology, Natural variation in plants, Plant development

## Abstract

The significance of automatic plant identification has already been recognized by academia and industry. There were several attempts to utilize leaves and flowers for identification; however, bark also could be beneficial, especially for trees, due to its consistency throughout the seasons and its easy accessibility, even in high crown conditions. Previous studies regarding bark identification have mostly contributed quantitatively to increasing classification accuracy. However, ever since computer vision algorithms surpassed the identification ability of humans, an open question arises as to how machines successfully interpret and unravel the complicated patterns of barks. Here, we trained two convolutional neural networks (CNNs) with distinct architectures using a large-scale bark image dataset and applied class activation mapping (CAM) aggregation to investigate diagnostic keys for identifying each species. CNNs could identify the barks of 42 species with > 90% accuracy, and the overall accuracies showed a small difference between the two models. Diagnostic keys matched with salient shapes, which were also easily recognized by human eyes, and were typified as blisters, horizontal and vertical stripes, lenticels of various shapes, and vertical crevices and clefts. The two models exhibited disparate quality in the diagnostic features: the old and less complex model showed more general and well-matching patterns, while the better-performing model with much deeper layers indicated local patterns less relevant to barks. CNNs were also capable of predicting untrained species by 41.98% and 48.67% within the correct genus and family, respectively. Our methodologies and findings are potentially applicable to identify and visualize crucial traits of other plant organs.

## Introduction

Species identification is a fundamental component in every discipline of biology to properly utilize, monitor, and protect highly diverse living organisms on Earth. However, conventional identification workflows that manually distinguish key visual features are slow and error-prone because of the complexity and intraspecific variation in morphological traits^1^. There is also increasing concern that the number of professional and amateur taxonomists is persistently declining, and that the gap in taxonomic knowledge between professionals and the public is increasing^1–3^. Thus, an increasing number of studies propose automated identification systems based on rapidly advancing machine learning methods, to support both professionals and the public^4–8^. Among the various fields where automation is being introduced, plant identification is one of the most vigorously studied areas with high demand owing to its easy accessibility, rich diversity, and increasing curiosity about natural creatures in urban life^9^.

In addition to conventional taxonomic approaches to identify plants, previous studies regarding automated plant identification have mainly focused on extracting visual features from either or both reproductive and vegetative organs^10–15^. However, the availability of these organs highly depends on the season, phenological changes, and height of the crown base, especially in tree species. Hence, owing to the non-rigid and three-dimensional nature of leaves and flowers, it is challenging to produce high-quality image data to train and evaluate machine learning models^16^. The challenges encountered in identification by leaves and flowers, have led several studies to exploit the benefit of using barks that persist throughout the season and are easy to access and process with their simple cylindrical shapes^17^.

Limited studies on bark identification have focused on the terminology and description of visual patterns in the inner and outer bark^18^. Whitmore^19^ and Yunus, et al.^20^ visually inspected tropical trees and broadly categorized the patterns (e.g., deep/shallow fissured, scaly, and laminate). Prior to technological breakthroughs, taxonomists utilized these little-known bark traits as an auxiliary means of species identification. Recently, Hadlich, et al.^21^ used a portable spectrometer to identify bark from tropical species. Other methodologies, utilizing Red, Green, Blue (RGB) channel images, approached the problem as multi-class texture classification task consisted of two consecutive phases: representation and classification^22^. Representation (feature extraction) is an abstraction process that extracts useful features from the labyrinthine texture of barks, while classification assigns the features into discrete class labels by minimizing the error function of a specific machine learning algorithm. Early attempts mainly took advantage of hand-crafted features, including Gabor filters^23^, SIFT^24^, local binary patterns^25^, and local ternary patterns^22^, which were followed by classification using conventional machine learning algorithms (e.g., k-nearest neighbors, support vector machines, and multi-layer perceptron). Recently, these human-designed feature representation workflows have been rapidly substituted by data-driven approaches in which machines find the most appropriate features. Regarding data-driven approaches, especially for large-scale image data, convolutional neural networks (CNNs) are the most commonly adopted algorithms that are superior to conventional ones^26^. Carpentier, et al.^27^ also showed that CNNs could identify 23 different tree species with > 90% accuracy using only small-patched images of barks, which is a highly challenging task even for professionals^24^.

Despite the powerful performance of CNNs, there is an inevitable trade-off between the accuracy and interpretability of the model^28,29^. Due to the complexity and the large number of parameters of CNNs that reach over 480 million in current state-of-the-art approaches^30,31^, it is almost impossible to review the individual parameters by tracing back the model’s output. Lack of interpretability and the ability to explain the model’s prediction and accuracy could lead to concerns regarding the legitimacy and mystery of the performance of CNNs. These concerns are intensified by its weak taxonomic foundation and fewer morphological characteristics^32^. Previous studies also mainly focused on the contribution to the quantitative aspects by raising accuracy in specific datasets rather than expanding qualitative knowledge, which can be useful for professionals in other disciplines.

In this aspect, we focus on discovering the qualitative features of how state-of-the-art computer vision machines successfully unravel highly sophisticated patterns of nature. We used the latest advances in computer vision, class activation mapping (CAM), which enabled us to access one of the nested groups of parameters (layers) in CNNs and interpret the output by highlighting the most influential (activated) regions in making the prediction^29,33,34^. We prepared a bark image dataset of 42 tree species and trained two well-characterized CNN architectures: the old, simple, and effective VGG-16 and the state-of-the-art, but with high architectural complexity, EfficientNet. After the models were successfully trained and validated, we extracted and generated saliency maps for the entire sample with respect to each model. We proposed a novel approach called CAM aggregation (“Materials and methods”; Fig. 7), that enables the unified representation of diagnostic features over the entire bark. The heat maps were manually reviewed and documented as diagnostic keys for each species, and the reliability and limitations of our proposed method were investigated.

More specifically, we aimed to answer the following research questions:

(1) Can machine-learning algorithms be applied to identify woody species using bark characteristics? What accuracy can be expected from different samples?

(2) How do two machine-learning CNN algorithms (VGG-16 and EfficientNet) compare?

(3) Does the interpretation methodology (i.e., CAM) reveal specific diagnostic features that are important for their successful application?

## Results

**Figure 1:**
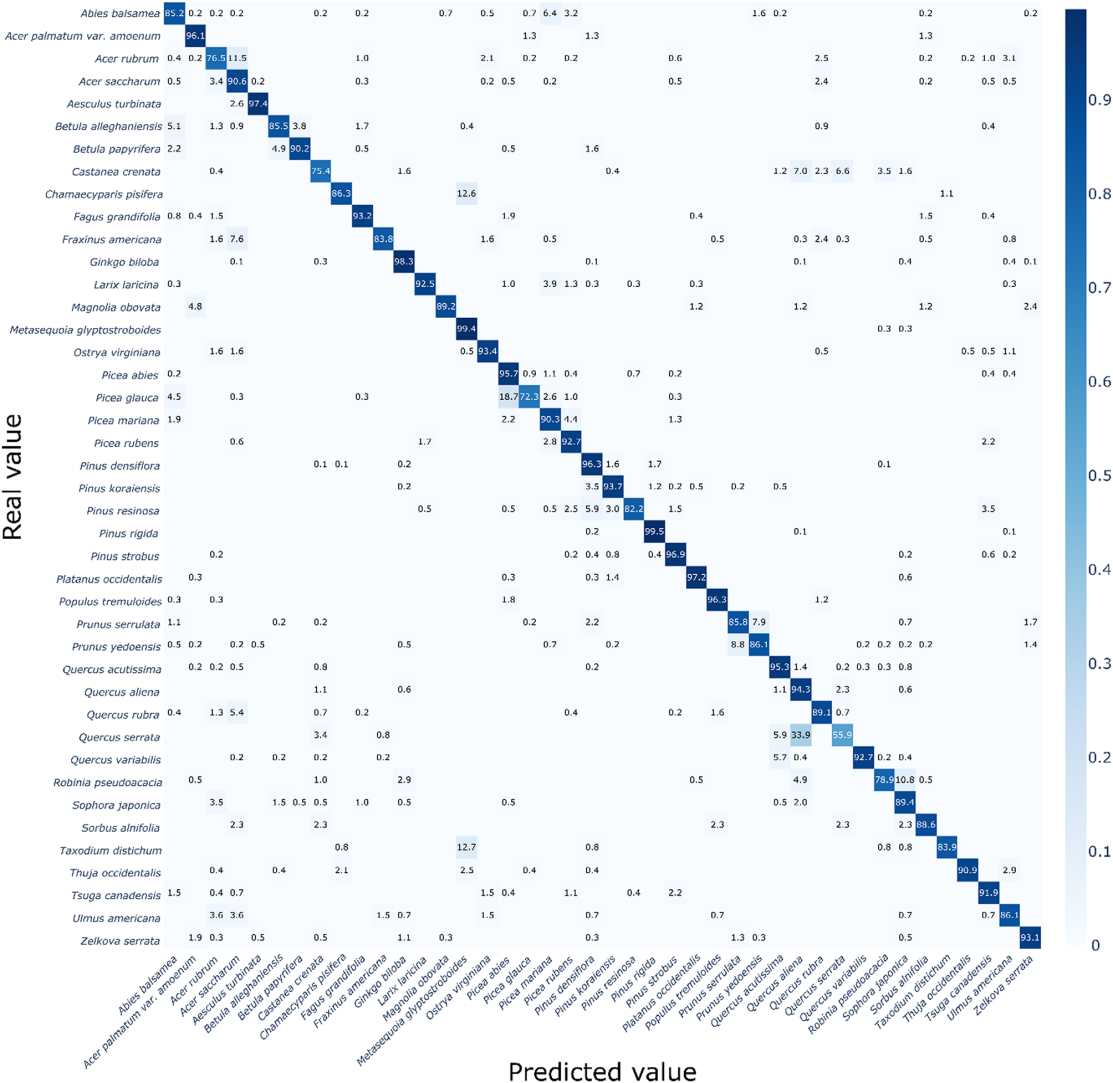
Confusion matrix of 42 species produced by VGG-16. The X- and Y-axis indicate the predicted class from the model and true label, respectively. The color of each cell represents the classification accuracy and actual accuracy values are written in each cell. Empty cells indicate the zero proportion, namely no prediction by the model. Detailed values for the figure are given in Supplementary data S1.

**Figure 2:**
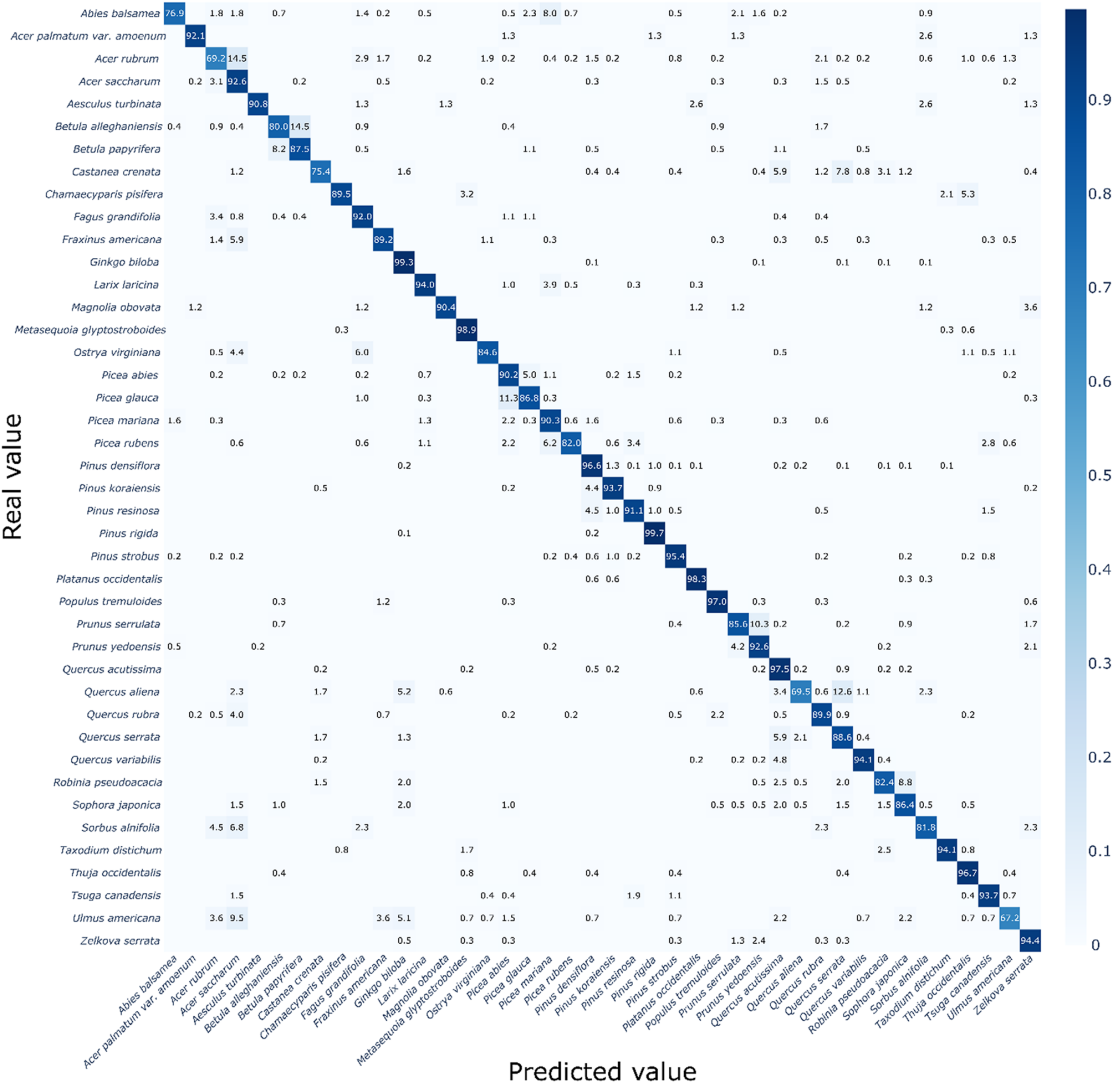
Confusion matrix of 42 species produced by EfficientNet. The X- and Y-axis indicate the predicted class from the model and true label, respectively. The color of each cell represents the classification accuracy and actual accuracy values are written in each cell. Empty cells indicate the zero proportion, namely no prediction by the model. Detailed values for the figure are given in Supplementary data S2.

The training of the two CNN models was successful, and both CNN models reached over 90% overall accuracy, as shown in Table 1. The performance metrics (“Materials and methods”) did not show substantial differences among the four measures, even between the models (ranged within 0.5%). A more complex model, namely EfficientNet, required a much larger amount of training to reach its maximum accuracy; it took 65 epochs in VGG-16 (90.7%) and 161 epochs in EfficientNet (91.0%). A more detailed comparison of classification performance with respect to each species is given as confusion matrices in Figs. 1 and 2, produced by VGG-16 and EfficientNet, respectively. Most species showed high accuracies of > 90%, but two models tended to show lower accuracies in some species that had multiple species in each genus or family, and they were mostly misclassified as other species in the same genus or family.

**Table 1:** Comparison of two trained CNN models with four performance metrics (“Materials and methods”).

	VGG-16 (%)	EfficientNet (%)
Overall accuracy	90.7	91.0
Precision_M_	89.6	90.5
Recall_M_	89.2	89.0
F-score_M_	0.89	0.90

VGG-16 and EfficientNet reached the best overall accuracy at 65th and 161st epoch, respectively.

In VGG-16, for example, large misclassifications were made in *Abies balsamea* (by 6.39% to *Picea mariana*), *Castanea crenata* (by 7.0% and 6.6% to *Quercus aliena* and *Quercus serrata*, respectively), *Taxodium distichum* (by 12.7% to *Metasequoia glyptostroboides*), *Acer rubrum* (by 11.5% to *Acer saccharum*), *Picea glauca* (by 18.7% to *Picea abies*), and *Quercus serrata* (by 33.9% to *Quercus aliena*). EfficientNet followed the same trend but showed weaknesses in slightly different species: *Abies balsamea* (by 8.0% to *Picea mariana)*, *Acer rubrum* (by 14.5% to *Acer saccharum*), *Betula alleghaniensis* (by 14.47% to *Betula papyrifera*), and *Quercus aliena* (by 12.6% to *Quercus serrata*). *Ulmus americana* was misclassified to species in other taxon groups, including *Acer saccharum* (9.5%) and *Ginkgo biloba* (5.1%).

Meanwhile, the accuracy of the CNN models for each species did not show a clear trend with the number of training samples (Figs. 1 and 2, and Table 2). Species with high error rates (e.g., *Acer rubrum*, *Castanea crenata*, *Picea glauca*, and *Quercus serrata*) were trained with an intermediate number of unique bark images, ranging from 161 to 226, whereas species trained with a relatively small number of images still showed high accuracies (e.g., *Acer palmatum* var. *amoenum*, *Magnolia obovata*, and *Picea rubens*). However, considering that the ImageNet dataset consisted of approximately 1000 images per class^35^, we suggest that more than 100 images per species should be prepared, assuming that 10 cropped pieces can be sampled for each bark image (Fig. 6, “Data pre-processing and augmentation”).

**Table 2:** Details of the tree species and corresponding number of bark images utilized in this study.

	Unique bark images	Cropped test samples
*Abies balsamea*^b^	151	438
*Acer palmatum* var*. amoenum*^a^	59	76
*Acer rubrum*^b^	226	477
*Acer saccharum*^b^	315	585
*Aesculus turbinate*^a^	63	76
*Betula alleghaniensis*^b^	134	235
*Betula papyrifera*^b^	104	184
*Castanea crenata*^a^	161	256
*Chamaecyparis pisifera*^a^	87	95
*Fagus grandifolia*^b^	148	264
*Fraxinus americana*^b^	226	370
*Ginkgo biloba*^a^	474	695
*Larix laricina*^b^	282	385
*Magnolia obovata*^a^	68	83
*Metasequoia glyptostroboides*^a^	305	351
*Ostrya virginiana*^b^	114	182
*Picea abies*^b^	285	461
*Picea glauca*^b^	175	310
*Picea mariana*^b^	176	318
*Picea rubens*^b^	97	178
*Pinus densiflora*^a^	774	1,230
*Pinus koraiensis*^a^	341	431
*Pinus resinosa*^b^	112	202
*Pinus rigida* × *taeda*^a^	558	868
*Pinus strobus*^a,b^	283	480
*Platanus occidentalis*^a^	252	360
*Populus tremuloides*^b^	230	328
*Prunus serrulata*^a^	349	458
*Prunus yedoensis*^a^	335	433
*Quercus acutissima*^a^	421	639
*Quercus aliena*^a^	119	174
*Quercus rubra*^b^	331	552
*Quercus serrata*^a^	185	236
*Quercus variabilis*^a^	344	560
*Robinia pseudoacacia*^a^	140	204
*Sophora japonica*^a^	125	198
*Sorbus alnifolia*^a^	71	44
*Taxodium distichum*^a^	81	118
*Thuja occidentalis*^b^	149	242
*Tsuga canadensis*^b^	170	270
*Ulmus americana*^b^	93	137
*Zelkova serrata*^a^	262	377
In total	9375	14,560

Only the number of cropped test samples are described because we concurrently generated train samples during the training phase (see “Data pre-processing and augmentation”), rather than making the cropped samples from the prior train dataset.

Superscripts ^a^ and ^b^ denote the source of the images; ^a^ represents the data collected in this study, and ^b^ from BarkNet 1.0 dataset.

These results suggest that CNNs can successfully identify most species; however, varying the extent of intra-genus and intra-family similarities could lead to low accuracy, and two CNNs might learn and utilize classification keys in different ways.

**Figure 3:**
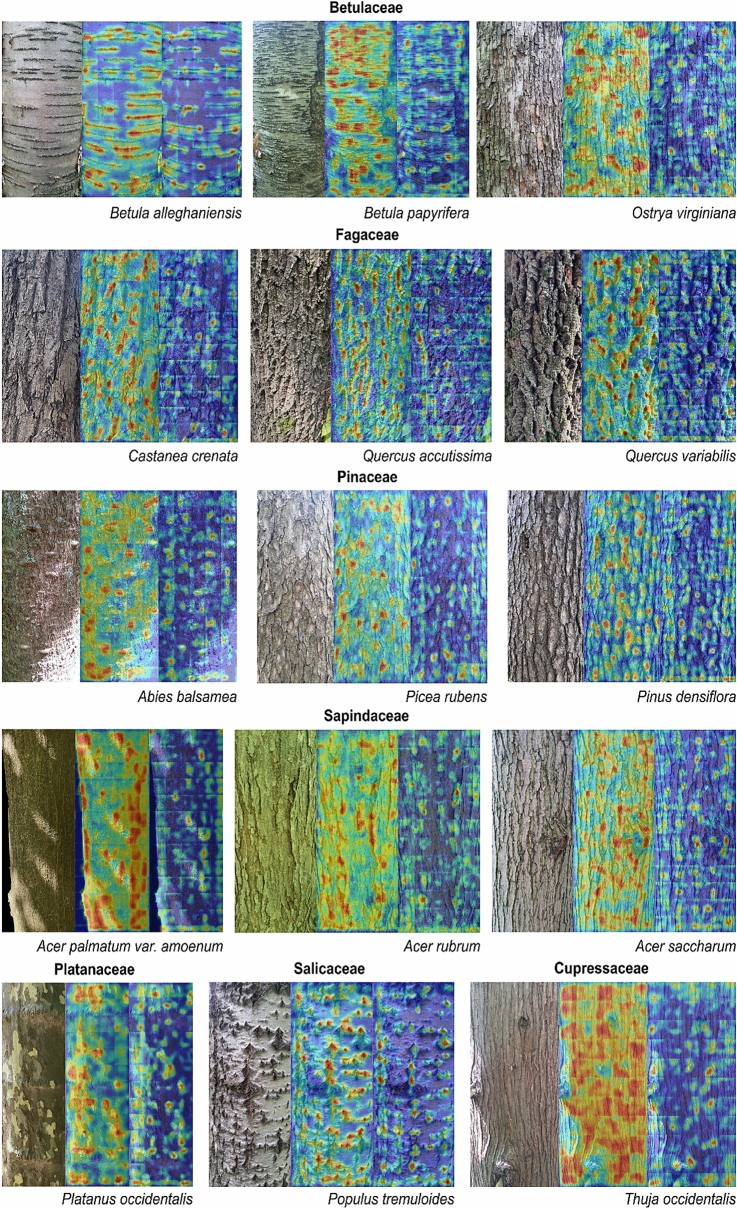
Representative CAM outputs from two CNN models; images on the left, middle, and right indicate the original image, CAM heatmap from VGG-16, and EfficientNet, respectively. Our proposed method, CAM aggregation (“Materials and methods”), enables to visualize unified representation of the diagnostic features over the entire bark. Regions highlighted in red denote the strongest activation from each model, which indicates the most relevant region for the prediction. CAM outputs of the entire 42 species are presented in the extended figures hosted on Figshare, https://doi.org/10.6084/m9.figshare.14727834.

The diagnostic features of each species were identified as heat maps displaying the relative importance of classification (Fig. 3 and extended figures at https://doi.org/10.6084/m9.figshare.14727834). In the heat maps extracted from VGG-16, the saliency responses in most species were typically matched with localized shape patterns, which were also readily noticeable by human eyes. For example, the patterns were shown as blisters in *Abies*, vertical stripes and crevices in *Acer*, large horizontal lenticels in *Betula*, shaded parts of scaly bark in *Picea*, scaly and flaking patterns in *Platanus*, small striped and dotted lenticels in *Prunus*, and vertical crevices and clefts in *Quercus* (detailed information by species listed in *SI Appendix,* Table S1). A large number of images was activated by higher density in several species that did not retain salient features but exhibited repeated textures along the entire bark. It was mostly found in four Cupressaceae species (*Chamaecyparis pisifera*, *Metasequoia glyptostroboides*, *Taxodium distichum*, and *Thuja occidentalis*) and in some samples of *Fagus grandifolia*, and *Magnolia obovata,* as large numbers of elongated narrow cracks or stripes. These results indicate that VGG-16 mostly catches locally prominent features if available, and if not, they utilize the entire texture patterns that are extensively shown and repeated.

In EfficientNet, the disparity of heat maps in terms of both quantity and quality was observed. The model utilized much smaller and weakly activated regions that did not match the proposed patterns from VGG-16 in many species. Along with smaller sizes of each activation region, they were less matched with the exact location of salient features including stripes, lenticels, and crevices, and also showed activation for some digressive and irrelevant features of barks (e.g., backgrounds, mosses, knots, leaves, and catkins) (Fig. 3 and extended figures at https://doi.org/10.6084/m9.figshare.14727834).

## Discussion

Our results suggest that CNNs have sufficient capacity to identify tree bark images with high accuracy in most species. However, the proposed methods also showed weaknesses in distinguishing the inter-species similarities between some genera and families, which might be attributed to disparity in diagnostic features, whereas the overall accuracy did not show substantial differences between the two models.

The contrasting diagnostic features from EfficientNet could be explained by its complicated architecture consisting of 54 convolution layers, 17 pooling layers, and other dropout and reshape layers (240 layers in total). In contrast, VGG-16 consists of 13 convolution layers and five pooling layers (23 layers in total). Convolution layers at different depths learn distinct levels of representation, and the deepest layers are trained to capture the features with the highest abstraction^36^. It could be inferred that increasing the number of convolution layers and architectural complexity led the CNN model to utilize more condensed information in the data.

In this aspect, augmenting the complexity of the model increased performances of large-scale image classification datasets (e.g., ImageNet) of objects with rigid boundaries and intermediate intra-class variances. However, natural images show large variation in the same species (age and environmental variation) and frequently show high inter-species similarity. If the model excessively focuses on the local features shown in a specific entity rather than grasping general patterns shared at the species level, it might overfit and decrease overall performance.

Previous studies reported that ImageNet pre-trained models showed high domain adaptation ability. That is, high accuracy models on ImageNet showed higher accuracies on other domains (R^2^ > 0.95), tested with a benchmark dataset consisted of 65 *objects* (Office-Home Dataset)^37^. In this study, EfficientNet did not show substantially better accuracy than VGG-16, regardless of the superior performance on ImageNet.

The varying accuracies and CAM representation capacities of CNNs with natural images have been studied less, but few studies are in accordance with the findings of this study. When tested for detecting flowering events using five distinctive CNN architectures^38^, the accuracies ranged only within 1.3%, but CAM visualization yielded largely contrasting results. In the study, simpler models showed activation to the large and relevant region to flowers, whereas the complex and better performing model showed much smaller activated region. Therefore, the complex model predicted flowering by only a small number of flowers with higher sensitivity.

In the case of barks, augmented complexity and sensitivity could have mixed effects on both positive and negative sides. Complex models might identify species with more localized and sophisticated features that simple models cannot find, but along with the risk of overfitting. Consequently, ensemble techniques that combine and predict different types of models or architectural modifications that enable multi-receptive levels (e.g., Inception modules in GoogLeNet^39^) would be suitable for real-world applications.

**Figure 4:**
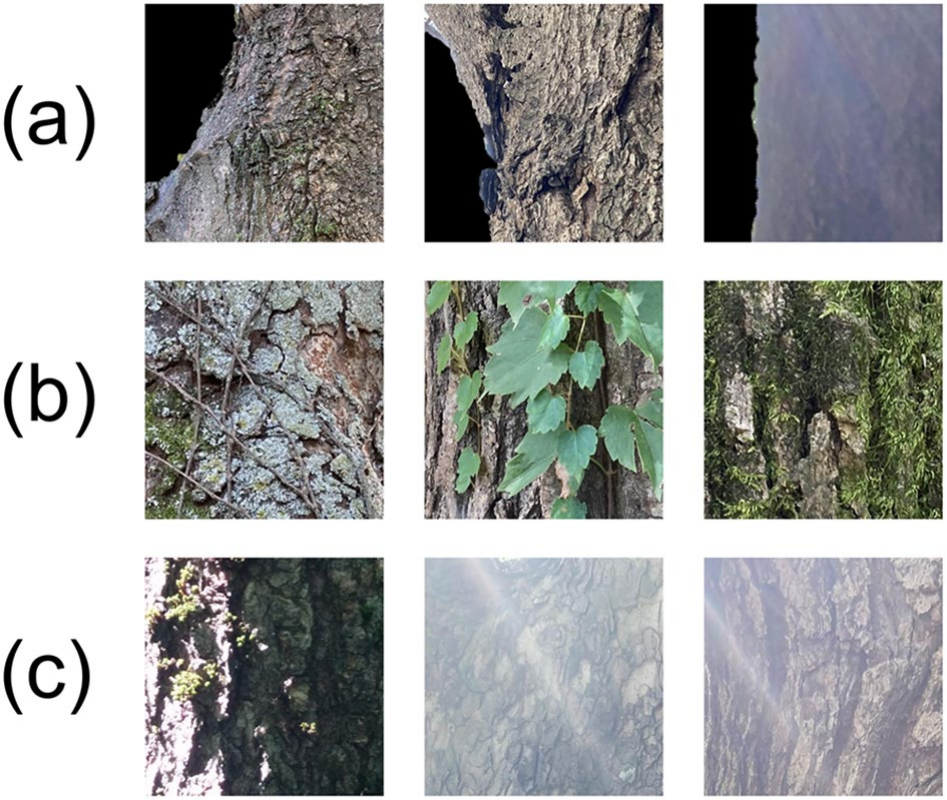
Bark image samples misclassified from the CNN models. (**a**–**c**) Represent typical conditions in which the CNN models showed the most errors.

A qualitative inspection of misclassified samples in Fig. 1 and 2 revealed that CNNs tended to show errors in some typical conditions which should be avoided in data preparation. They could be typified as excessive background masking in Fig. 4a, foreign object coverage in Fig. 4b, and improper light conditions in Fig. 4c. The other misclassifications that could not be typified, may originated from high inter-species similarity along with the black-box characteristics of deep learning models. As shown by Rzanny, et al.^14^, where fusing multiple organs increased accuracy up to 20%, combining the bark with leaf and flower characteristics would lead to improved accuracy and robustness.

**Figure 5:**
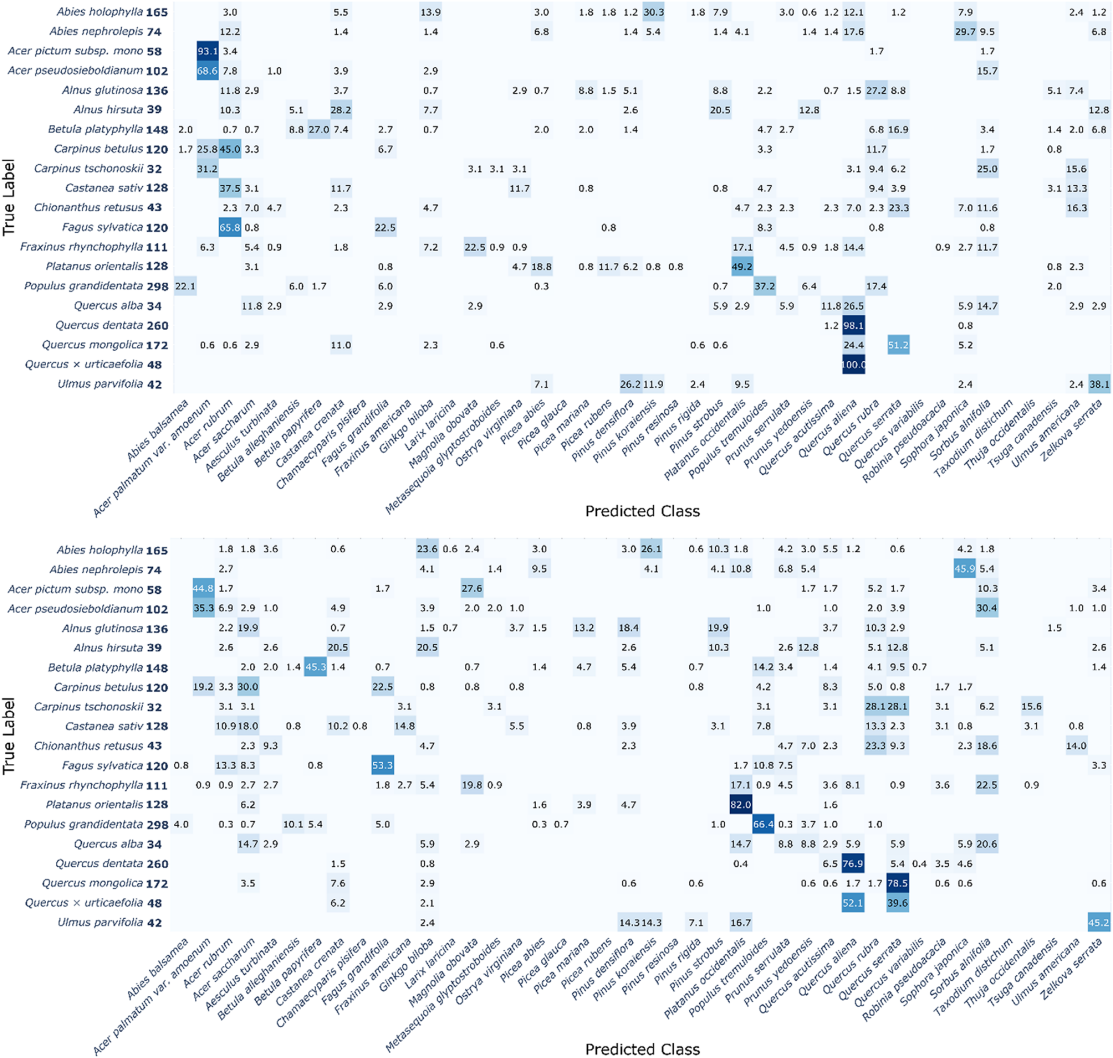
Confusion matrix produced by two models, VGG-16 (above) and EfficientNet (below), using the image data of untrained species, excepted due to the insufficient number of samples to be trained as a discrete class. The X-axis denotes the prediction class from the original dataset (42 species), and Y-axis indicates the true label from the additional untrained dataset (20 species). The numbers in the Y-axis labels indicate the number of tested samples of each species. Detailed values for the figure are given in Supplementary data S3.

Meanwhile, CNN models tend to misclassify other species in the same genus or family (Figs. 1 and 2). These results imply that diagnostic features are shared within higher taxa, and CNNs can recognize and utilize these general features for identification. We further investigated this generalization capacity with images of new species within the same genus or family^27^, consisting of 20 species in *Abies*, *Acer*, *Alnus*, *Betula*, *Carpinus*, *Castanea*, *Chionanthus, Fagus*, *Fraxinus*, *Platanus*, *Populus*, *Quercus*, and *Ulmus* (Fig. 5). When tested with two models, VGG-16 predicted 42.0% of the total samples to the species in the same genus (among 42 original species), and 48.7% to the species in the same family; and EfficientNet predicted 37.8% and 44.5% of the species in the same genus and family, respectively. In detail, VGG-16 showed high generalization ability in *Acer pictum* subsp. *mono* (93.1%) and *Acer psuedosieboldianum* (68.6%) into *Acer palmatum* var*. amoenum*, *Platanus orientalis* (49.2%) into *Platanus occidentalis*, *Quercus dentata* (98.1%) and *Quercus* × *urticaefolia* (100%) into *Quercus aliena*. EfficientNet particularly showed better results in *Betula platyphylla* (45.3%), *Fagus sylvatica* (53.3%), *Platanus orientalis* (82.0%) and *Populus grandidentata* (66.4%). These results suggest that CNNs can catch the shared features within a higher taxon and categorize new species into their genera or families if trained before. Furthermore, it seems that VGG-16 learned and utilized more generalized features from the original dataset than EfficientNet, which showed more localized and digressive diagnostic feature representations (Fig. 3 and extended figures at https://doi.org/10.6084/m9.figshare.14727834).

In summary, we demonstrated the capacity of CNNs to distinguish sophisticated patterns in barks and proposed a novel approach to extract and narrate diagnostic keys found by computer vision models. We insist that architectural differences in CNNs largely affect the quality of diagnostic features, which is not shown by quantitative accuracy metrics. Our proposed methods are easily applicable to other plant organs (e.g., leaves, flowers, and wood anatomical features) that also exhibit complex patterns on their surfaces. Furthermore, we suggest that using the bark as a baseline prediction factor and combining the information from other organs which will enable highly precise tree species identification.

## Materials and methods

### Bark image datasets

The Bark dataset for training and evaluation of CNNs was prepared by combining two different datasets: BarkNet 1.0^27^ and the additional dataset collected for this study. In terms of size and number of species, BarkNet is currently the only publicly available dataset of its kind and contains more than 23,000 bark images captured from 23 tree species found in Quebec City, Canada, with 10–40 repeated captures from different angles and heights of individual trees. The captured raw images were pre-processed in the dataset by two discrete crop processes: trimming off two vertical border lines between the trunk and the background (side-crop) and then cropping into square-shaped patches to match the input shape for CNNs (square-crop). In this study, side-cropped images of the BarkNet dataset were utilized. As the images in BarkNet were captured with a large number of repetitions, this might lead to high train–test dataset similarity, which impedes efficient training of CNNs. Consequently, four images were randomly sampled from each of 10 to 40 repetitions and then manually filtered out of images that share a large portion of each other.

For the additional dataset, the images were captured according to the protocols of BarkNet with minor modifications. The protocols were as follows: captured from September to November 2020; in parks and forests near Seoul City, Republic of Korea; with a wide range of crown density; under various weather conditions from sunny to cloudy and rainy; using an iPhone SE2 camera; at a distance between 20 and 60 cm away from the trunk; with three to four repetitions from different angles per individual tree. The species selected were common species that can be easily found in most parks and forests in the Republic of Korea. We collected samples from multiple species in each genus to investigate intra-genus similarities.

Consequently, two datasets consisting of 3672 images (20 species; BarkNet 1.0) and 5703 images (23 species; for the present study) were prepared, having one species *Pinus strobus* in common. A more detailed list of species and the corresponding number of captured images in the two datasets are given in Table 2.

### Data pre-processing and augmentation

**Figure 6:**
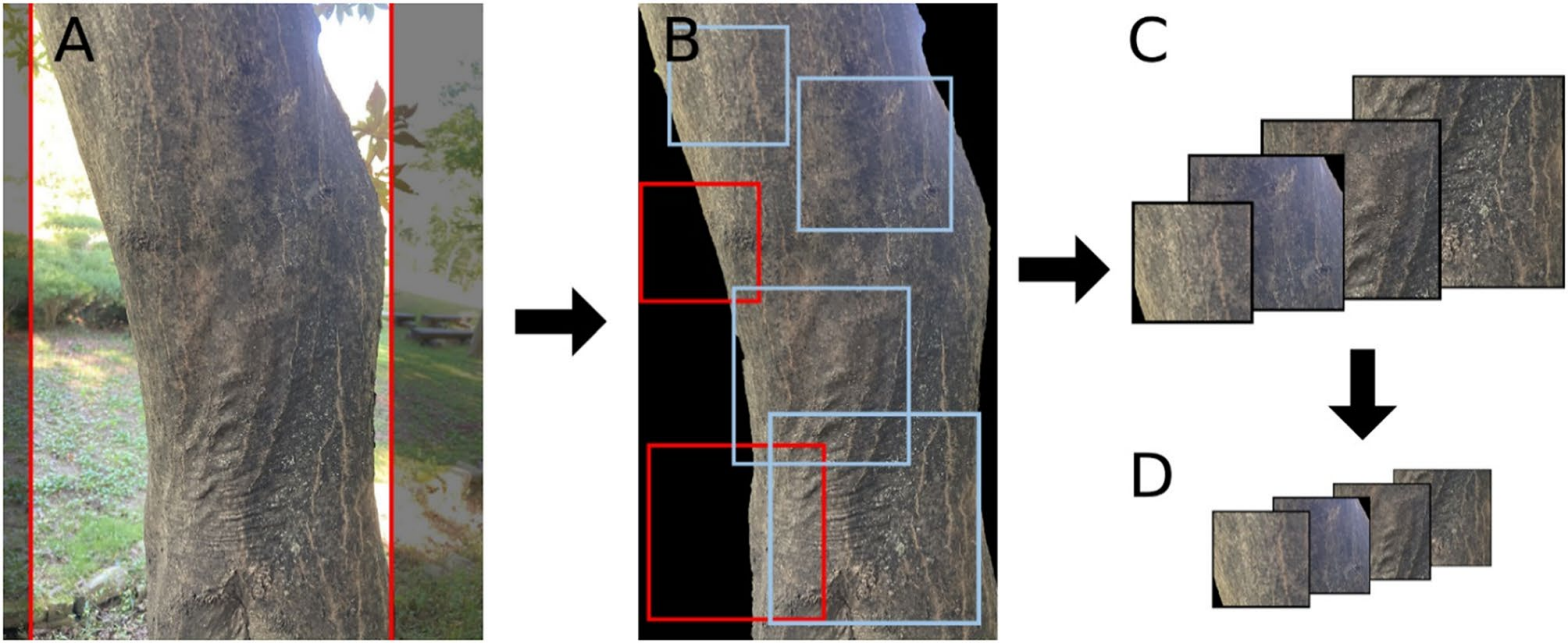
Demonstration of data pre-processing procedures. Raw images were side-cropped to a length that did not cut out the bark (**A**) and the backgrounds were masked with black pixels (**B**). Then, the images were sampled using random window sizes (40–60% of total width) and position (**B**). If the window contained more black pixels than the pre-defined threshold (0.05 in the present study), the window was re-sampled. After the batched crop images were prepared (**C**), they were resized to match the model input size and ‘RandAugment’ was applied (**D**). These procedures were performed concurrently during the training phase.

The overall pre-processing and augmentation are illustrated in Fig. 6. Before aggregating the two datasets, side-crop and masking were applied to our dataset. Because our dataset contained images that had non-vertical boundary lines, all images were side-cropped to a length that did not cut out pixels of barks, and the remaining background was masked by black pixels. After the images were cropped and masked, BarkNet and our dataset were aggregated and split into two subsets along with each class: 80% and 20% for training and testing, respectively.

For the training dataset, a random square crop with thresholding was applied to the images before putting them into the CNNs. Unlike previous studies, the images were randomly square-cropped by proportional length instead of fixed pixel lengths, such as 224 × 224^27^ and 256 × 256^40^. For each crop, the pixel length of the crop was first randomly selected in the range of 40–60% of the total width, and then the position of the cropping square was randomly sampled. To exclude excessive background, the ratio of masked pixels to total pixels was calculated, and if the ratio was higher than the threshold (0.05, in this study), the crop region was re-sampled. This approach has several advantages: minimizing the loss of bark features that overlap with crop region, flexible cropping that copes with varying resolutions, and cropped images that require no manual filtering.

For data augmentation, a process that increases the training data by giving slight modifications, ‘RandAugment’ was utilized^41^. RandAugment is an automated data augmentation process that randomly selects a predefined number of augmentation functions and then applies them with a randomly sampled magnitude from a predefined range. The ‘candidate’ functions include equalization, rotation, solarization, adjustment of contrast, brightness, and color. The entire list of augmentation functions and hyperparameters regarding RandAugment could be found in the provided Python scripts (“Materials and methods”: Data and code availability).

These random square-crop and augmentation processes were applied concurrently with the training. More specifically, side-cropped training samples remained unmodified, the random square-crop followed by augmentation was performed, and the output images with the number of predefined batch sizes were fed into the CNNs. For the testing dataset, each side-cropped sample was square-cropped by 50% of the total width, without randomized sampling, and no augmentation was applied. All training and testing samples were resized to 331 × 331 before data augmentation.

### Convolution neural networks

In this study, among numerous CNN architectures that have been extensively studied and released, the following architectures were adopted and compared: VGG-16 and EfficientNet^42,43^. As one of the early CNN architectures released in 2014, VGG-16 remains a widely used CNN architecture due to its simple structure and powerful performance in various computer vision tasks, including classification^44^, detection^45^, and segmentation^46^. In contrast, EfficientNet is currently the most powerful CNN architecture, showing the highest benchmark accuracy on ImageNet (26) while reducing the number of parameters compared to other recent models. EfficientNet is an approach that searches for the best scaling coefficients in terms of the width, depth, and resolution of CNNs. In this study, EfficientNet with the smallest scale size (B0) was used to match the input size with VGG-16.

Using two CNN architectures, each model was modified for effective transfer learning by following sequences: (1) truncate the final fully-connected (FC) layer which contained neurons for predicting 1000 classes (in the original ImageNet dataset), (2) reshape (flatten) the output of the final convolution layer and apply dropout, (3) insert a new FC layer with the same number of neurons as the prediction classes (42 species), (4) apply softmax activation with cross-entropy loss functions.

Here, a convolution layer performs element-wise multiplication between the input data and the weights; a pooling layer simply reduces the dimensions of the input data; a dropout layer randomly sets input node values to zero and prevents overfitting; and a FC layer connects all nodes in one layer to every node in itself.

### Extracting diagnostic features with class activation mapping aggregation

Since CAM was first proposed by Zhou, et al.^33^, there have been several advances in CAM techniques. The original CAM had certain limitations in that it required a global average pooling layer in the architecture, along with laborious re-training. These limitations were overcome by Selvaraju, et al.^29^ in gradient-weighted class activation mapping (Grad-CAM), using a back-propagation gradient in any target convolution layer. An advanced method called Grad-CAM++ has recently been proposed, which provides better localization capability than Grad-CAM, especially with multiple object instances occurring in a single image. Considering that the diagnostic features would show multiple occurrences in a single bark image, Grad-CAM++ was utilized in this study as the CAM extraction method.

**Figure 7:**
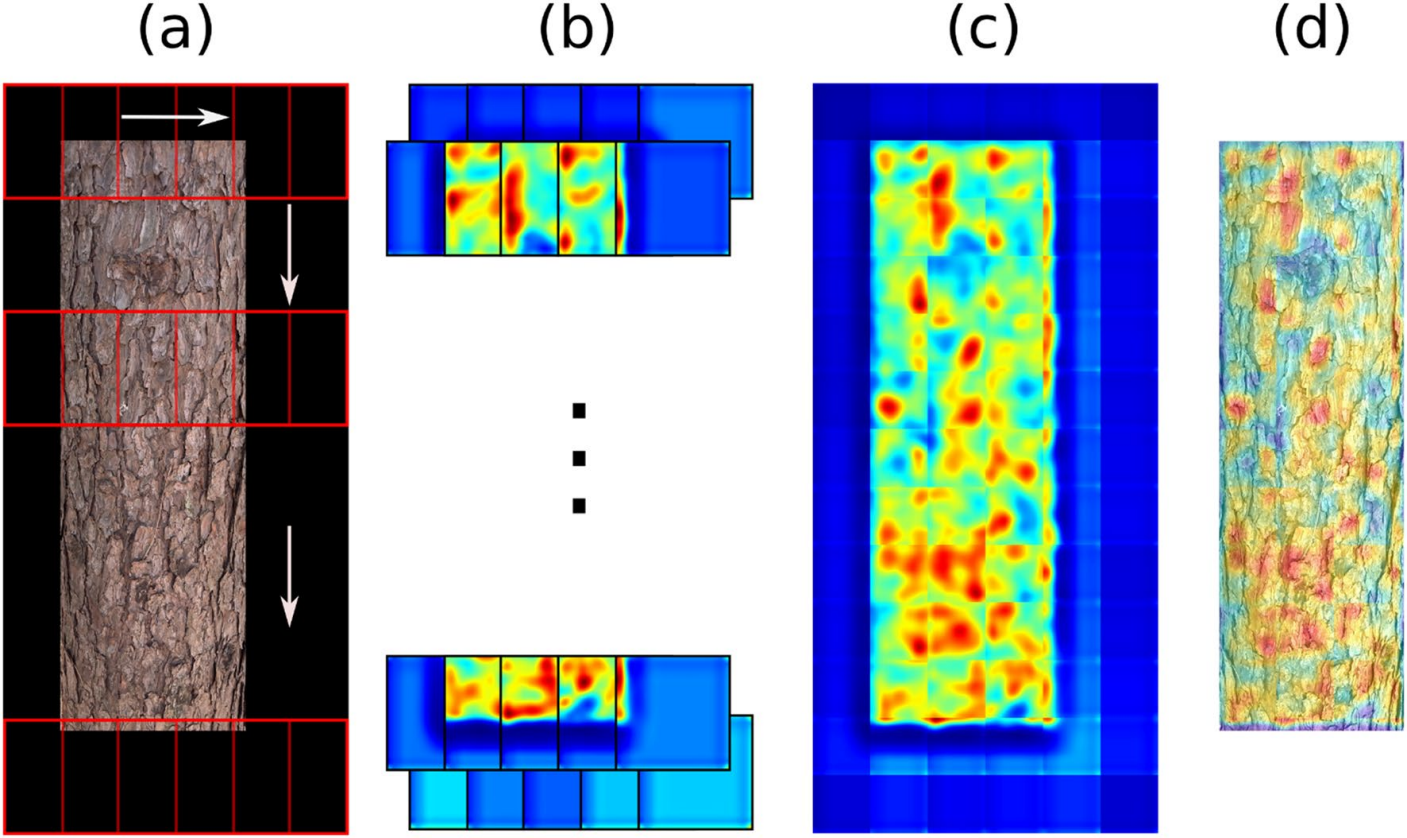
Demonstration of the consecutive process of CAM aggregation. The black background and red-bordered squares in (**a**) indicate the zero-padded pixels and sliding windows, respectively. The patched heatmaps in (**b**) represent the CAM outputs of the corresponding window, calculated from the last activation layer of each model. (**c**) Aggregated and averaged CAM outputs over the entire bark. (**d**) Superimposed image of the heatmap to the original image.

To build a general representation of diagnostic features over the entire bark rather than individual square-cropped patches, aggregation and averaging methods were applied, referred to as CAM aggregation (Fig. 7). The CAM of each tree image was calculated using the following steps: zero-padding, CAM calculation from sliding windows, and taking the mean of CAM outputs. More specifically, bark images of whole trees were padded with black pixels with corresponding widths and heights so that the window slides over the bark area for an equivalent number of times. Then, the receptive window slid over the padded image with a predefined stride of half window size and calculated the CAM of the cropped image. All CAM calculations were then combined and averaged along the entire bark image.

### Performance metrics

Two CNN models were evaluated using four performance metrics: overall accuracy and macro-averaged precision, recall, and f-score with $\upbeta$ = 1. Each metric was calculated by the multi-class classification performance measurements adopted from Sokolova and Lapalme^47^, using overall accuracy instead of average accuracy in this study. The measurements were as follows:

$$\mathrm{Overall\,accuracy}=\sum\limits_{\mathrm{i}=1}^{\mathrm{l}}\frac{{\mathrm{tp}}_{\mathrm{i}}}{{\mathrm{tp}}_{\mathrm{i}}+{\mathrm{tn}}_{\mathrm{i}}+{\mathrm{fp}}_{\mathrm{i}}+{\mathrm{fn}}_{\mathrm{i}}}$$

$${\mathrm{Precision}}_{\mathrm{M}}=\frac{{\sum }_{\mathrm{i}=1}^{\mathrm{l}}\frac{{\mathrm{tp}}_{\mathrm{i}}}{{\mathrm{tp}}_{\mathrm{i}}+{\mathrm{fp}}_{\mathrm{i}}}}{\mathrm{l}}$$

$${\mathrm{Recall}}_{M}=\frac{{\sum }_{i=1}^{l}\frac{t{p}_{i}}{t{p}_{i}+f{n}_{i}}}{l}$$

$$\mathrm{F}-\mathrm{scor}{e}_{M}=\frac{\left({\upbeta }^{2}+1\right)Precisio{n}_{M}Recal{l}_{M}}{{\upbeta }^{2}Precisio{n}_{M}+Recal{l}_{M}}$$

where $t{p}_{i}$, ${\text{tn}}_{i}$, ${fp}_{i}$, and $f{n}_{i}$ represent true positive, true negative, false positive, and false negative counts, respectively, for each species class ${\mathrm{C}}_{\mathrm{i}}$ and with $\mathrm{l}$ number of total classes ($\mathrm{l}=42$). $\mathrm{M}$ indices represent the macro-averaged calculation of each metric.

### Training details

When training VGG-16 and EfficientNet, we utilized the following hyper-parameters: 10^−5^ as the learning rate, Adaptive Moment Estimation (Adam) as the optimizer, 50% for dropout rate of the fully connected layer, and training batch size of 8. Two CNN models were pre-trained with the ImageNet dataset, and then fine-tuned until the maximum overall accuracy on test dataset did not increase for 10 consecutive epochs. CNN models were trained and tested under Windows 10 pro 64-bit OS and Python version 3.8.5, with hardware specification of an AMD Ryzen 5 3600XT CPU, 32 GB of RAM and an NVIDIA GTX 1080 Ti GPU. Tensorflow (2.4.1) and Keras (2.4.3) were utilized as deep learning frameworks.

## Supplementary Information

Supplementary Information 1.

Supplementary Information 2.

Supplementary Information 3.

Supplementary Information 4.

## Data Availability

The BarkNet 1.0 dataset was retrieved from https://github.com/ulaval-damas/tree-bark-classification^27^; the bark image data collected in this study were published and are available on Zenodo (10.5281/zenodo.4749062)^48^.

## Code availability

The python scripts used in this study are available on GitHub (https://github.com/snutp/TBKFE). Refer to the scripts in the repository for technical implementation details of the methods in this study.
