# Supplementary material for: Identifying and extracting bark key features of 42 tree species using convolutional neural networks and class activation mapping
Source: Sci Rep. 2022 Mar 19;12:4772. doi: 10.1038/s41598-022-08571-9 (PMC8934343; doi:10.1038/s41598-022-08571-9)
Supplement: Supplementary file 4 — Supplementary Information 4. [file 41598_2022_8571_MOESM4_ESM.docx]

# Supplementary Tables

**Table S1. Morphological and diagnostic bark features of 42 tree species. Diagnostic keys were illustrated by manually reviewing the CAM outputs from VGG-16. Refer to the extended heatmap representations hosted on Figshare,** [**doi.org/10.6084/m9.figshare.14727834**](https://doi.org/10.6084/m9.figshare.14727834)**.**

| Species | Color | Textures | Diagnostic Keys |
| --- | --- | --- | --- |
| ***Abies balsamea*** | Ash, grayish brown, red-brown | Blisters, smooth, scaly and flaking circular bark plates, dotted lenticels | Blisters, lenticels, red-brown inner barks, shadowed parts of scales |
| ***Acer palmatum* var*. amoenum*** | Gray with bright stripes | Smooth; long vertical stripes | Long, bright, and vertical stripes |
| ***Acer rubrum*** | Light gray, gray-brown | Long and vertical shallow furrows and fissures | Vertical ridges, vertical shallow fissures |
| ***Acer saccharum*** | Light gray, gray-brown, dark gray furrows | Long and vertical shallow furrows and fissures, deep fissures and clefts | Vertical and horizontal fissures, clefts |
| ***Aesculus turbinata*** | Dark gray, light brown | Smooth, irregular red-brown vertical stripes repeated | Repeated textures with red-brown vertical stripes |
| ***Betula alleghaniensis*** | White, gray | Smooth or peeling, Long and narrow lenticels | Horizontal lenticels, bark peels |
| ***Betula papyrifera*** | White, black patches | Smooth or peeling; long, narrow, and vertically crossing lenticels | Horizontal lenticels with vertical crossing lines |
| ***Castanea crenata*** | Gray, dark brown | Deep x-shaped furrows, clefts, irregular horizontal cracks | Deep fissures, clefts, flat vertical ridges |
| ***Chamaecyparis pisifera*** | Red-brown, light gray, orange-brown | Long vertical peeling, repeated shallow furrows | Shadows of flaking barks and vertical furrows |
| ***Fagus grandifolia*** | Light gray | Smooth, small dotted to long vertical dark repeated stripes, dotted lenticels | Repeated textures with dotted and vertical dark stripes |
| ***Fraxinus americana*** | Gray, red-brown inner barks | Shallow x-shaped repeated furrows, small clefts | Vertical and horizontal furrows, clefts |
| ***Ginkgo biloba*** | Gray, dark patches along the furrows | Irregular deep furrows, clefts | Not clear |
| ***Larix laricina*** | Gray, bright red inner barks | Scaly, flaky, irregular small cracks | Shadows of irregular bark cracks |
| ***Magnolia obovata*** | Gray, dark patches | Smooth, narrow vertical dark repeated stripes and cracks, dotted lenticels | Repeated vertical stripes and cracks |
| ***Metasequoia glyptostroboides*** | Dark gray, light brown inner barks | Long and vertical shallow peeling and cracks | Colored inner barks opened by cracks and peels, vertical cracks |
| ***Ostrya virginiana*** | Light gray, red-brown | Flaking long vertical barks, long parallel repeated cracks | Shadows of vertical and horizontal fissures |
| ***Picea abies*** | Gray, dark green, bright red inner barks | Scaly and flaking irregular bark plates, smooth inner barks | Shadows of irregular bark scales |
| ***Picea glauca*** | Bright red, dark scales | Smooth or scaly and flaking circular bark plates | Shadows of circular bark scales |
| ***Picea mariana*** | Light gray, red-brown inner barks | Scaly and flaking irregular bark plates | Shadows of irregular bark scales and cracks |
| ***Picea rubens*** | Light gray, red-brown inner barks | Smooth or scaly and flaking irregular bark plates | Shadowed parts of irregular bark scales and cracks |
| ***Pinus densiflora*** | Ash, red-brown inner barks | Long, vertical, large bark scales; deep furrows | Long vertical ridges |
| ***Pinus koraiensis*** | Ash, light gray, bright red inner barks | Long, vertical, irregular bark scales; flaking | Shadowed and bright flaky bark scales |
| ***Pinus resinosa*** | Ash, dark green, bright red inner barks | Long, vertical, irregular bark scales; irregular deep furrows | Shadows of bark scales and deep furrows |
| ***Pinus rigida* × *taeda*** | Dark gray, light gray | Irregular deep crevices, clefts | Shadows of clefts and horizontal crevices |
| ***Pinus strobus*** | Gray, bright red inner barks | Shallow furrows and cracks | Shadows of vertical furrows and cracks, long vertical ridges |
| ***Platanus occidentalis*** | Gray, green and white inner barks | Small, flaky bark scales; smooth inner barks | Bright colored patches of smooth inner barks, flaking bark plates |
| ***Populus tremuloides*** | White, dark gray fissures, red-brown inner barks | Smooth; short to long vertical fissures; | Wide and short to long vertical fissures, rhombus shaped fissures |
| ***Prunus serrulata*** | Gray, dark and light brown lenticels | Smooth; long, horizontal lenticels with small brown colored dotted parts; | Long horizontal lenticels |
| ***Prunus yedoensis*** | Gray, dark and light brown lenticels | Smooth; long horizontal lenticels with small brown colored dotted parts; | Light brown dotted lenticels, long horizontal lenticels |
| ***Quercus acutissima*** | Dark gray, light brown inner barks | Vertical and parallel x-shaped furrows, shallow clefts | Long vertical furrows, shadows of shallow clefts |
| ***Quercus aliena*** | Gray, dark gray | Irregular vertical furrows, shallow clefts | Shadows of vertical furrows and clefts |
| ***Quercus rubra*** | Light gray, bright red | Smooth, white vertical stripes and furrows; | White vertical stripes and furrows, ridges near the deep furrows |
| ***Quercus serrata*** | Gray, red-brown inner bark | Irregular vertical furrows | Shadows of vertical furrows and clefts, ridges near the furrows |
| ***Quercus variabilis*** | Gray | Irregular vertical deep x-shaped furrows deep clefts | Shadows of deep vertical furrows and clefts |
| ***Robinia pseudoacacia*** | Light gray | Vertical and parallel ridges overlapped with x-shaped patterns, shallow to deep furrows | Long vertical ridges, shadowed parts of deep vertical furrows |
| ***Sophora japonica*** | Light to dark gray | Long, vertical, shallow furrows and flat ridges | Shadows of vertical furrows, flat shaped ridges |
| ***Sorbus alnifolia*** | Ash, dark gray | Smooth or vertical shallow fissures, irregular dark stripes | Repeated texture with dark stripes |
| ***Taxodium distichum*** | Gray-brown, red-brown | Long, vertical, shallow furrows; peeling | Not clear |
| ***Thuja occidentalis*** | Red-brown | Long, vertical, shallow furrows; peeling | Repeated textures of vertical furrows |
| ***Tsuga canadensis*** | Gray-brown, red-brown | Smooth, wide vertical ridges and furrows | Long vertical ridges |
| ***Ulmus americana*** | Ash, light gray, red-brown inner barks | Long, vertical, shallow furrows | Colored inner barks opened by vertical shallow and deep furrows |
| ***Zelkova serrata*** | Ash, dark gray, red-brown lenticels | Scaly and flaking irregular bark plates, dotted or horizontal lenticels | Repeated texture of horizontal lenticels, shadowed parts of bark scales |
